# Construction and validation of a predictive model for postoperative urinary retention after lumbar interbody fusion surgery

**DOI:** 10.1186/s12891-023-06816-w

**Published:** 2023-10-13

**Authors:** Dong Tian, Jun Liang, Jia-Lu Song, Xia Zhang, Li Li, Ke-Yan Zhang, Li-Yan Wang, Li-Ming He

**Affiliations:** 1 Department of Orthopaedic Surgery, Shanxi Bethune Hospital, Shanxi Academy of Medical Sciences, Taiyuan, China; 2 Third Hospital of Shanxi Medical University, No. 99, Longcheng street, Taiyuan city, 030032 Shanxi Province China; 3 Tongji Shanxi Hospital, Taiyuan, China

**Keywords:** Predictive model, Postoperative, Urinary retention, Lumbar interbody fusion

## Abstract

**Background:**

Postoperative urine retention (POUR) after lumbar interbody fusion surgery may lead to recatheterization and prolonged hospitalization. In this study, a predictive model was constructed and validated. The objective was to provide a nomogram for estimating the risk of POUR and then reducing the incidence.

**Methods:**

A total of 423 cases of lumbar fusion surgery were included; 65 of these cases developed POUR, an incidence of 15.4%. The dataset is divided into a training set and a validation set according to time. 18 candidate variables were selected. The candidate variables were screened through LASSO regression. The stepwise regression and random forest analysis were then conducted to construct the predictive model and draw a nomogram. The area under the curve (AUC) of the receiver operating characteristic (ROC) curve and the calibration curve were used to evaluate the predictive effect of the model.

**Results:**

The best lambda value in LASSO was 0.025082; according to this, five significant variables were screened, including age, smoking history, surgical method, operative time, and visual analog scale (VAS) score of postoperative low back pain. A predictive model containing four variables was constructed by stepwise regression. The variables included age (β = 0.047, OR = 1.048), smoking history (β = 1.950, OR = 7.031), operative time (β = 0.022, OR = 1.022), and postoperative VAS score of low back pain (β = 2.554, OR = 12.858). A nomogram was drawn based on the results. The AUC of the ROC curve of the training set was 0.891, the validation set was 0.854 in the stepwise regression model. The calibration curves of the training set and validation set are in good agreement with the actual curves, showing that the stepwise regression model has good prediction ability. The AUC of the training set was 0.996, and that of the verification set was 0.856 in the random forest model.

**Conclusion:**

This study developed and internally validated a new nomogram and a random forest model for predicting the risk of POUR after lumbar interbody fusion surgery. Both of the nomogram and the random forest model have high accuracy in this study.

## Introduction

Lumbar interbody fusion (LIF) is a common surgical method for the treatment of lumbar degenerative disease. Catheterization is required during LIF due to the long operative time and the potential for bleeding. Traditional open posterior lumbar interbody fusion (PLIF) is a effective surgery and has some disadvantages, including trauma, excessive bleeding and iatrogenic low back pain [1]. In recent years, minimally invasive surgery (MIS) has developed rapidly, and MIS transforaminal lumbar interbody fusion (MIS-TLIF) and endoscopic lumbar interbody fusion (Endo-LIF) are representative techniques that have the advantages of reducing intraoperative bleeding and iatrogenic low back pain [2, 3]. Each of the above surgical methods has its advantages and disadvantages, which are widely used in clinic. Recent studies supports early catheter removal after surgery to reduce the risk of urinary tract infection [4]. However, patients have a risk of POUR after the catheter removal. Most patients with POUR need recatheterization, and this increases the risk of urinary tract infection(12.1%) and may ultimately prolong the length of hospitalization(POUR 1.9 days, non-POUR 0.9 days, p < 0.001. POUR 5.8 + 3.3 vs. non-POUR 4.9 + 3.9 days) and increase the hospitalization cost(POUR: $3,418.63; non-POUR: $2,681.43, p < 0.001) [5–7].

POUR is a very common complication after lumbar surgery, and its reported incidence is 15.4–25.9% in different studies [5, 7–11]. However, the methods used in these studies are not uniform. For example, the inclusion criteria and the diagnostic criteria for POUR differed in each study (Table 1). Previous studies have reported that age, sex, obesity, operative time, fusion surgery, delayed ambulation, postoperative thoracic epidural analgesia, transfusion volume, high VAS score, opioids, glonbromide, history of urinary retention, benign prostatic hyperplasia, and urinary tract infection are risk factors for POUR [5, 7–14]. In Chang’s study, a total of 31,251 patients (POUR = 2,858, no POUR = 28,393) were included in the meta-analysis. The incidence of POUR after spinal surgery was 15.1%. Being elderly, being male, having benign prostatic hyperplasia, having diabetes, and having a history of urinary tract infection are risk factors for POUR. Longer operative time and increased transfusion volume also increase the risk of POUR [12].

**Table 1:** Participant Characteristics

	[ALL]N = 416	Normal N = 358	Intubation N = 58	*P*
Sex, n (%)				0.208
Male	229(55.0%)	202(56.4%)	27(46.6%)	
Female	187(45.0%)	156(43.6%)	31(53.4%)	
Age, y	59.0[49.0;67.0]	58.0[48.0;67.0]	63.0[57.0;68.0]	0.009
BMI	24.2[22.3;26.6]	24.4[22.3;26.7]	24.2[21.7;25.3]	0.396
Smoke, n (%)				0.002
No	292(70.2%)	262(73.2%)	30(51.7%)	
Yes	124(29.8%)	96(26.8%)	28(48.3%)	
Hypertension, n (%)				0.643
No	301(72.4%)	261(72.9%)	40(69.0%)	
Yes	115(27.6%)	97(27.1%)	18(31.0%)	
Hemoglobin, g/L	138[126;148]	138[126;148]	138[126;144]	0.466
Thrombocyte, *10.9/L	214[173;258]	211[173;259]	234[175;252]	0.250
Scr, umol/L	70.8[62.2;79.6]	70.8[62.5;79.6]	70.2[61.2;80.2]	0.900
BUN, mmol/L	5.30[4.47;6.70]	5.40[4.40;6.70]	5.10[4.50;6.35]	0.344
ALB, g/L	39.8[37.5;41.8]	40.1[37.5;41.9]	38.9[36.3;41.6]	0.123
UA, umol/L	314[270;367]	317[269;367]	310[272;361]	0.768
Serum Potassium, mmol/L	3.94[3.78;4.14]	3.94[3.77;4.11]	3.96[3.78;4.32]	0.428
Operation method, n (%)				0.020
Endo-LIF	121(29.1%)	113(31.6%)	8(13.8%)	
MIS-TLIF	40(9.62%)	34(9.50%)	6(10.3%)	
PLIF	255(61.3%)	211(58.9%)	44(75.9%)	
Operative time, Minitue	180[150;220]	180[150;210]	240[180;300]	< 0.001
VAS of leg pain				
preoperation	3.00[3.00;3.00]	3.00[3.00;3.00]	3.00[3.00;3.00]	0.652
postoperation	2.00[1.00;2.00]	2.00[1.00;2.00]	2.00[2.00;2.00]	0.006
VAS of back pain				
preoperation	3.00[3.00;3.00]	3.00[3.00;3.00]	3.00[3.00;3.00]	0.931
postoperation	2.00[1.00;2.00]	2.00[1.00;2.00]	2.00[2.00;2.00]	< 0.001

Scr: serum creatinine. BUN: blood urea nitrogen. ALB: Serum albumin. UA: Serum uric acid.Endo-LIF: Endoscopic lumbar interbody fusion. MIS-TLIF: Minimally invasive surgery lumbar interbody fusion. PLIF: Posterior lumbar interbody fusion

Knowledge of specific risk factors has limited clinical value for determining whether a patient will develop POUR. A clinical prediction model can quantify the risk of POUR and provide a more intuitive and powerful scientific tool for clinical prevention. Few studies have proposed prediction models for POUR after lumbar surgery. Porche et al. constructed a sensitive prediction model using a combination of regression and neural network modeling. The identified predictors included diabetes, abnormal heartbeat, altered mental status, and screening for cardiovascular disorders. All of the included patients in this study underwent lumbar surgery, but the type of surgery was not specified. There may be large errors in different application scenarios.

The study reported in this paper included patients who underwent LIF (PLIF, MIS-TLIF, or Endo-LIF) surgery. A database containing as much detail as possible was established. based on this information, A nomogram for predicting the risk of POUR after LIF was constructed and validated. In addition, this study constructed the random forest model for comparision. The objective of the study is to identify patients with a high risk of POUR using a predictive model.

## Methods

### Study design

The study described here is a retrospective cohort study of POUR after LIF surgery. The study was approved by the Institutional Review Board (YXLL-2023-098). The methods and reporting guidelines proposed in the TRIPOD (Transparent Reporting of a multivariable prediction model for Individual Prognosis Or Diagnosis) statement were followed [15], as were the ethical principles set forth in the Declaration of Helsinki. Written informed consent was obtained from all participants. The study was performed at a single center, and the surgeries were performed by a single group of surgeons.

This study was conducted in the following order: (1) a predictive model for the occurrence of POUR after LIF surgery was developed; and (2) the model was validated.

### Participants

The inclusion criteria were as follows: ① more than 18 years of age; ② underwent LIF surgery, including PLIF, MIS-TLIF, and Endo-LIF; ③ a urinary catheter was placed after anesthesia and before surgery; and ④ the catheter was extracted 1–2 days after surgery. Individuals with ① abnormal urination due to cauda equina injury prior to surgery or ② urinary system disease indicators such as urinary hesitancy, poor stream, nocturia or treatment of prostatic hypertrophy with alpha agonists or ③ POUR caused by iatrogenic never damage were excluded from the study.

Data obtained from Shanxi Behune Hospital were used for the development and validation of the prediction models. The hospital is a provincial tertiary hospital in China and affiliated to Shanxi medical university.

Briefly, patients who underwent LIF surgery from January 2021 to June 2022 were included. The available data for this cohort included data abstracted from electronic medical records, including demographics, laboratory results, perioperative results, and VAS scores.

The dataset was split by time. We used the first phase (2021.1–2021.12) for model derivation and the second phase (2022.1–2022.06) for model validation. This approach has been shown to be methodologically more rigorous than a simple random split of the dataset [16, 17].

### Study outcome

The outcome was the development of POUR after LIF surgery.

### Definition of POUR after LIF surgery

POUR was defined as a ‘painful, palpable or percussible bladder, when the patient is unable to pass all urine [18] or post-void residual > 100ml [19]. All patients were allowed to ambulate with the waist, and recatheterization was performed if the patient was still unable to pass all urine.

### Retrieval of data

The electronic and digital medical record systems of the hospital were used to collect data according to the designed table. Two nurses input and analyzed the data. Another nurse checked the data and the outcomes to ensure the accuracy of the data.

### Statistical analysis

R software version 3.3.1 (The R Foundation for Statistical Computing, Vienna, Austria) was used in the statistical analysis.

Candidate predictor variables. LASSO regression was used to screen the variables. The optimal lambda was determined as the minimum lambda value plus the value of the standard deviation [20]. This value was used to screen for statistically significant predictors.

Multivariable discovery. We performed stepwise regression modeling using the Akaike information criterion and then applied Bayesian model averaging (BMA) to optimize model performance by both forward and backward selection [21, 22].

Missing data. Patients with missing data were omitted using the na.omit() function.

Random forest model. The parameters were as follows :ntree = 500, mtry = 3, and other parameters were the default values(randomForest package V.4.6–14). The Gini index was used as an impurity function. The details of the R package algorithm refer to the previous study by Biau and Scornet [23].

Performance of the prediction model. The performance of the nomogram was assessed by discrimination and calibration [24]. The discriminative ability of the model was determined by the AUC of the ROC curve, which ranged from 0.5 (no discrimination) to 1 (perfect discrimination) [25]. Calibration of the prediction model was performed using a visual calibration plot that compared the predicted and actual probabilities of POUR.

## Outcomes

### Participants

**Fig. 1:**
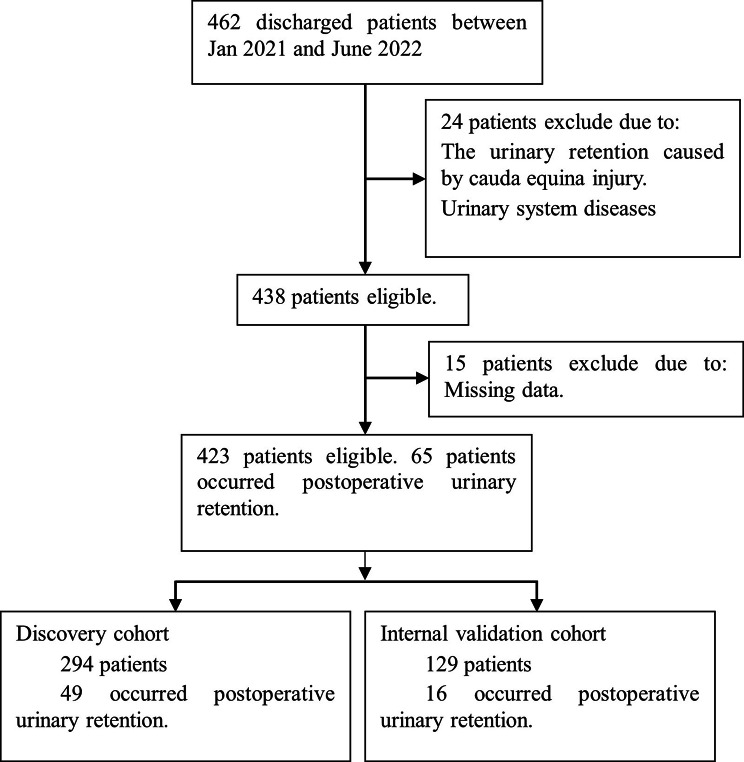
Flow chart of participants

A total of 462 patients were included from January 2021 to June 2022. 24 cases were excluded according to the exclusion criteria, and 15 cases with missing data were excluded. A total of 423 cases were finally included; among these cases, the incidence of POUR was 15.4% (65/423). A total of 294 patients from January 2021 to December 2021 were used as the training set to construct the model; of these, 49 had POUR, an incidence of 16.7%. A total of 129 patients from January 2022 to June 2022 were used as the validation set for model validation; of these patients, 16 had POUR, an incidence of 12.4%. The Flow chart is shown in Fig. 1. The participant characteristics are shown in Table 1.

### Model development

**Fig. 2:**
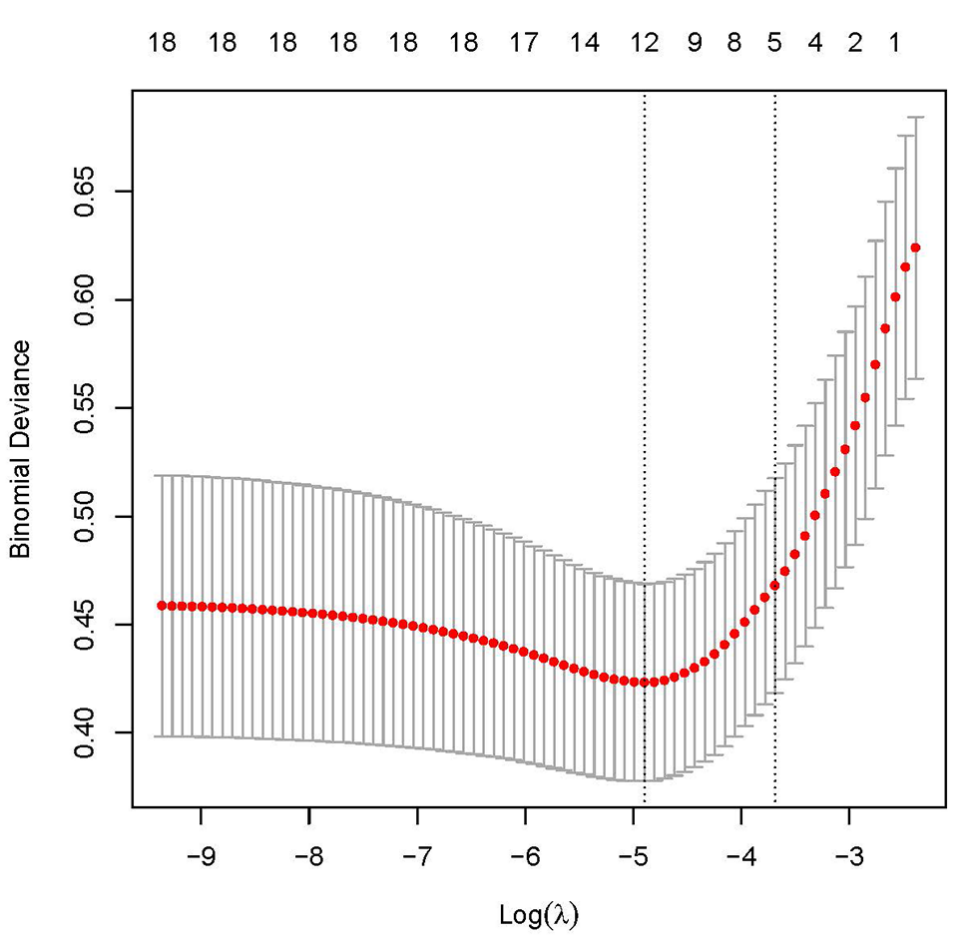
Log(lambda)

In this study, the regularization technique LASSO analysis was used to screen variables to effectively avoid overfitting. Through LASSO analysis, the best lambda value was determined to be 0.025082 (Fig. 2). Age, smoking history, operation method, operative time, and VAS score of low back pain were found to be significant predictors (Table 2).

**Table 2:** The outcomes of LASSO regression in training set

Variables	All (N = 288)	Normal (N = 245)	Intubation (N = 43)	*P*
Age, y	61.0[50.0;68.0]	60.0[48.0;68.0]	64.0[57.0;68.0]	0.027
Smoke, n (%)				0.001
No	205(71.2%)	184(75.1%)	21(48.8%)	
Yes	83(28.8%)	61(24.9%)	22(51.2%)	
Operation method, n (%)				0.038
Endo-LIF	82(28.5%)	76(31.0%)	6(14.0%)	
MIS-TLIF	33(11.5%)	29(11.8%)	4(9.30%)	
PLIF	173(60.1%)	140(57.1%)	33(76.7%)	
Operative time, Minitue	180[150;240]	180[150;210]	240[180;290]	< 0.001
VAS of low back pain (postoperation)	2.00[1.00;2.00]	2.00[1.00;2.00]	2.00[2.00;2.00]	< 0.001

The number of events per variable (EPV) was 9.8 in the logistic regression stage. Age, smoking history, operation method, operative time, and VAS score of low back pain were used in the stepwise regression (both forward and backward methods). The operation method was eliminated (β = 0.047, OR = 1.048). Smoking history (β = 1.950, OR = 7.031), operative time (β = 0.022, OR = 1.022) and VAS score of low back pain (β = 2.554, OR = 12.858) were statistically significant predictors (Table 3).

**Table 3:** Presenting the Prediction Model

Variables	β	SE	Wald χ^2^	*P*	OR	OR 95%CI	
						Lower	Upper
Age	0.047	0.018	6.777	0.009	1.048	1.012	1.086
Smoke	1.950	0.457	18.184	0.000	7.031	2.869	17.231
Operative time	0.022	0.004	28.663	0.000	1.022	1.014	1.030
VAS of low back pain (postoperation)	2.554	0.605	17.829	0.000	12.858	3.929	42.074
Intercept	-14.697	2.175	45.677	0.000	0.000		

**Fig. 3:**
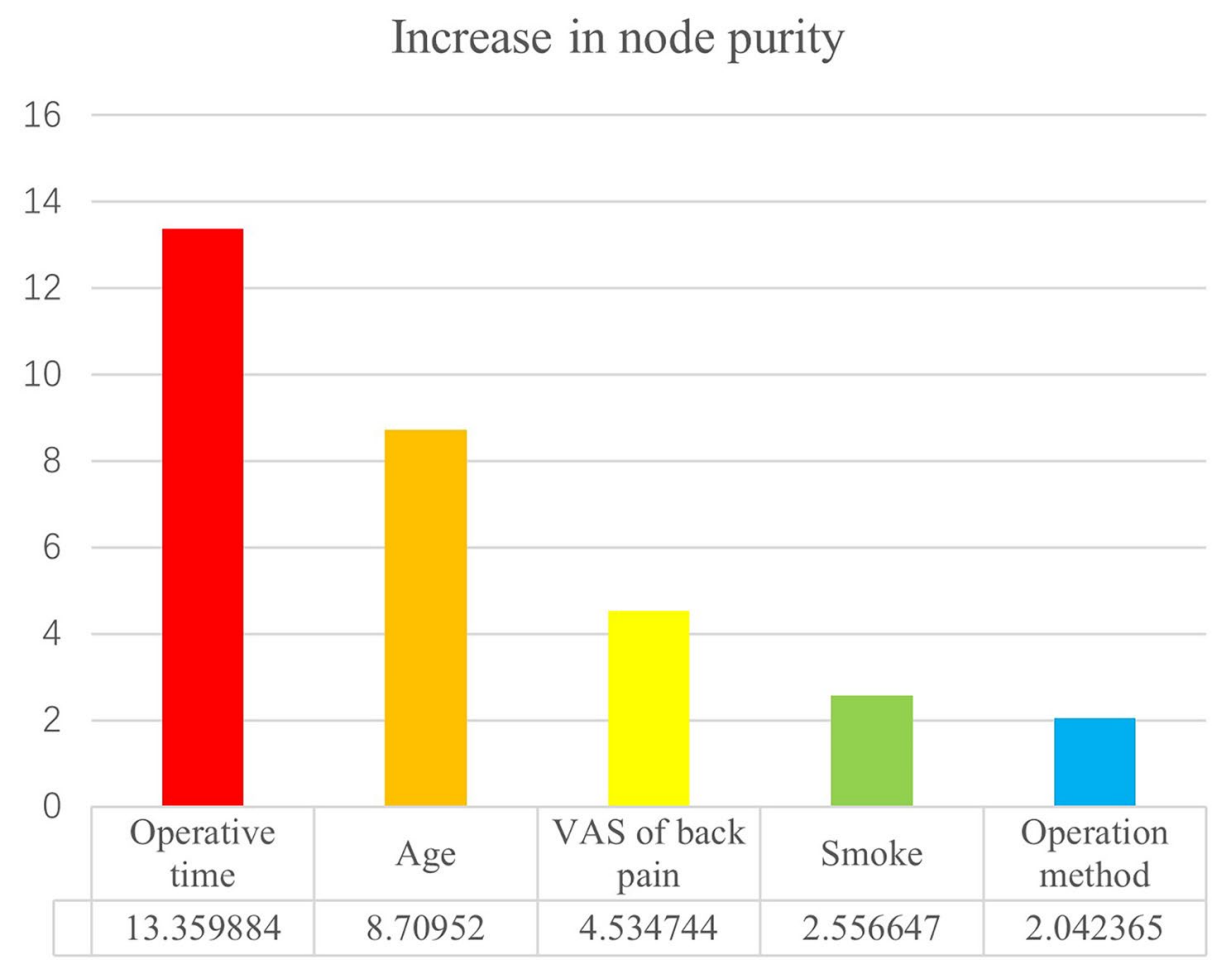
The increase in node purity

In addition, Age, smoking history, operation method, operative time, and VAS score of low back pain were chosen into the random forest model. The increase in node purity and the importance of variables was in the Fig. 3.

### Predictive nomogram for the probability of POUR

**Fig. 4:**
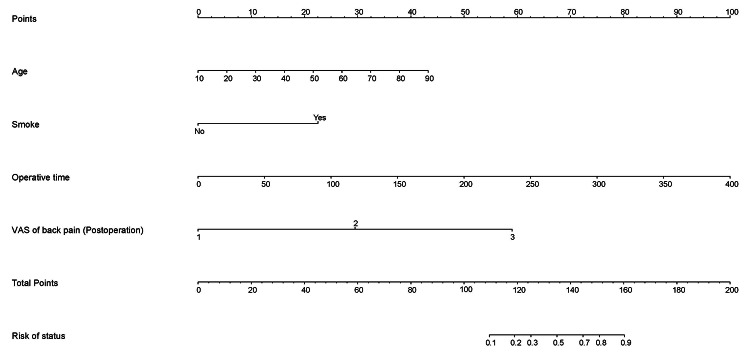
A nomogram, and how to use it to obtain a predicted probability in an individual

According to the results of stepwise regression, a nomogram including four statistically significant predictors was drawn (Fig. 4). A score was assigned for each predictor based on the upper scale, and the total score was calculated by adding these individual scores. The risk of POUR was estimated by projecting the total score to the lower scale of risk of status.

### Performance of the model

**Fig. 5:**
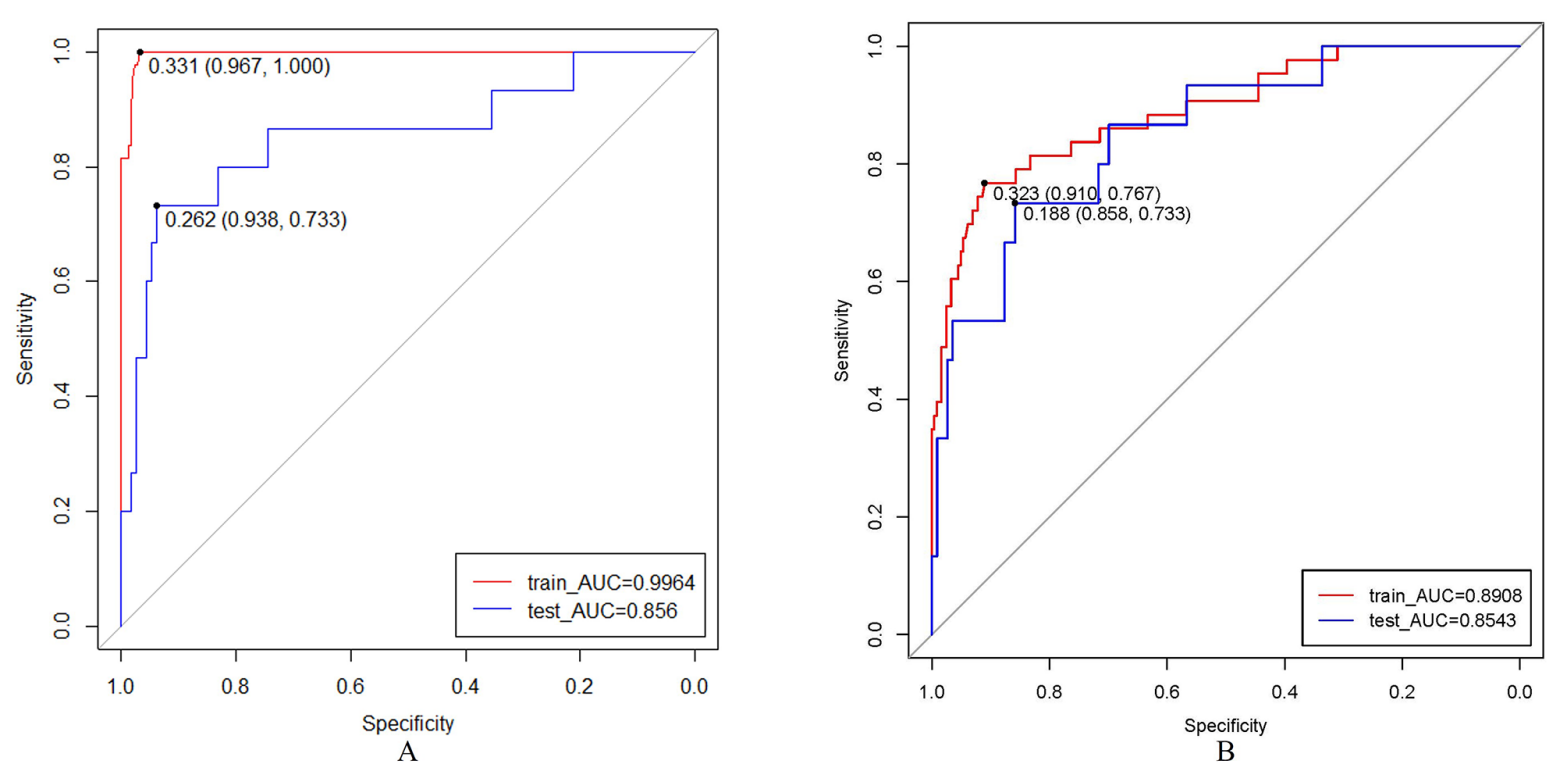
The area under the curve (AUC) of the receiver operating characteristic (ROC). (**A**) The stepwise regression model. (**B**) The random forest model

Based on ROC analysis, the nomogram shows strong discrimination. The AUC of the training set was 0.891, and that of the verification set was 0.854 in the stepwise regression model (Fig. 5A). The AUC of the training set was 0.996, and that of the verification set was 0.856 in the random forest model (Fig. 5B).

**Fig. 6:**
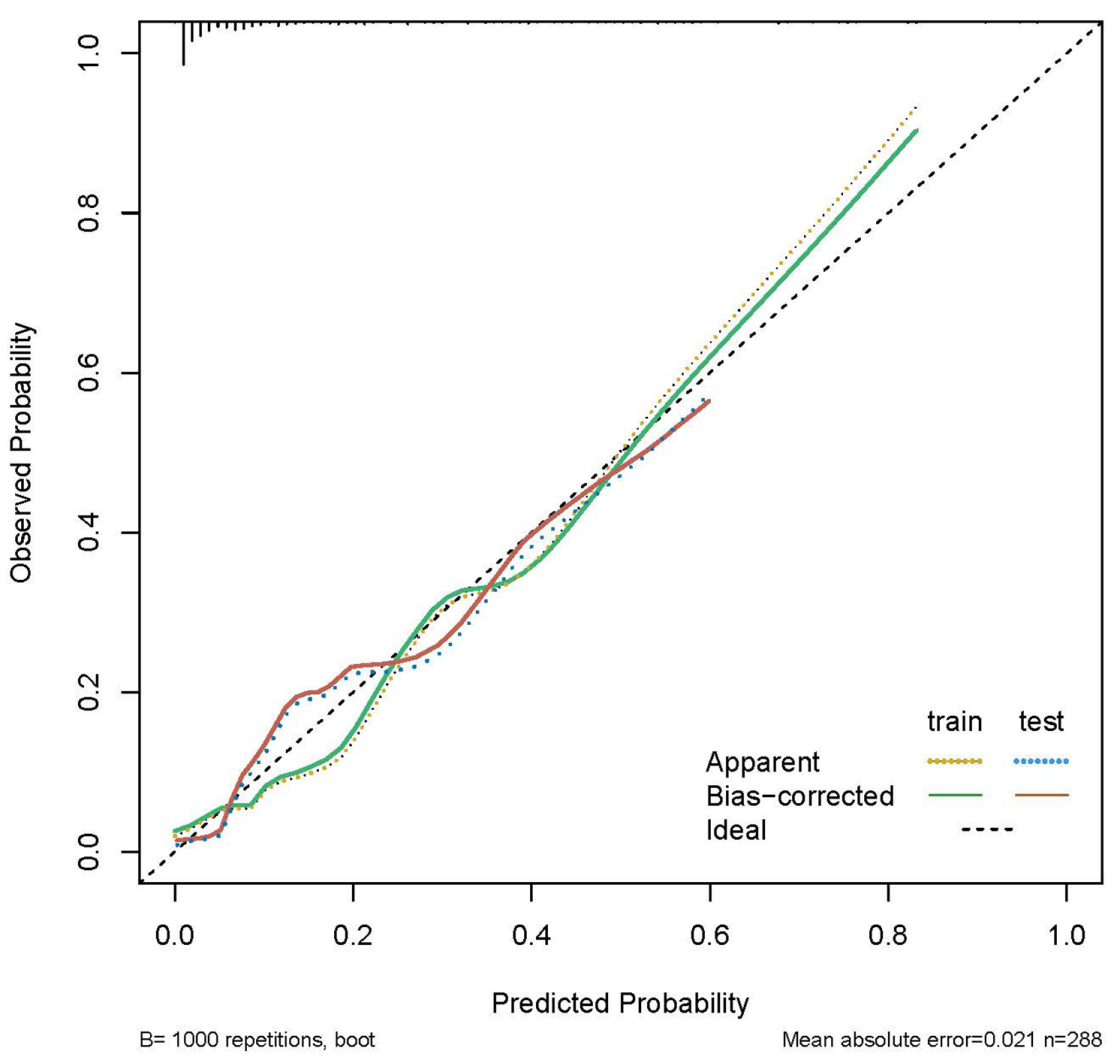
Calibration curves demonstrating the performance of the stepwise regression model

The calibration curve of the nomogram is shown in Fig. 6. The probabilities predicted by the nomogram for the training and validation sets matched the actual probability satisfactorily.

## Discussion

### Limitations

The model presented here for predicting POUR after LIF has many limitations. First, the sample size was not strictly calculated, and this may have led to overfitting due to the small sample size. In this study, 18 variables were included in the preliminary screening, and the EPV was low at this time. After preliminary screening of the variables, the EPV when entering the stepwise regression model was 9.8. The overall number of positive events was less than 100. These factors will affect the stability of the model; however, we also used corresponding methods to compensate for these shortcomings. Second, as many variables as possible should be included in the first-step analysis. However, Some variables that are closely related to the results may not have been included, such as anesthetic, mental status, infusion volume, et al. Third, this study is a retrospective cohort study. Thus, unlike in a prospective cohort study, the collected data could not be controlled in advance; this likely led to some deficiencies in the accuracy of the data, and some interesting information could not be obtained. This may affect the reliability of the predictive model. Lastly, this study is a single-center study and lacks an external validation set, and this may affect the universal applicability of the model; thus, further study is needed to complete the external validation.

### Interpretation

There is no research on predictive models for POUR after LIF. Porche et al. constructed a predictive model to predict the risk of urine retention after lumbar surgery. The research subjects were patients who had received lumbar surgery, but the study did not describe the specific types of surgery. A prediction model might be invalid in other scenarios if the composition of the sample in terms of the types of surgery they received is quite different. To improve the accuracy of the predictive model and avoid such limitations on the application scenario, this study limited the research subjects to patients undergoing LIF. This can improve the model accuracy and is valuable in research involving small sample sizes.

Although as many variables as possible were included in this study, there was a lack of information on mental status, infusion volume, and previous history of urinary tract infection. The reason for this is as follows. The assessment of mental status is subjective, and outcomes may differ significantly for this reason. In addition, this type of data cannot be acquired in a retrospective study. The infusion volume after LIF is relatively stable, and there is little difference among patients with respect to infusion volume. There were few patients with previous urinary tract infections in our study group.

The variables in this study included general data, perioperative data and laboratory examination outcomes. Five variables (age, smoking history, surgical method, operative time and VAS score of low back pain) were statistically significant predictors according to LASSO regression. The reason that surgical methods appeared as significant predictors may be that different surgical methods require different operative times, resulting in an overlap of this variable with the operative time variable. Through further stepwise regression, surgical method was eliminated from the list of statistically significant predictors. The final model included age, smoking history, operative time, and VAS score of low back pain. These predictors are basically consistent with those identified in previous studies on risk factors for postoperative urinary retention.

In this study, the AUC and the calibration curve were used to evaluate the prediction ability of the predictive model. The the stepwise regression model showed good prediction ability in the training set (0.891) and in the validation set (0.854); both of these values are close to the “good” level [26] [27]. The probabilities predicted by the nomogram in the training set and the validation set matched the actual probability satisfactorily. The coefficient of determination (R^2^) obtained in this study is 0.433.

This study constructed the random forest model for comparison with the stepwise regression model. The random forest model was better than the stepwise regression model in the AUC of the training sets. However, there was no significant difference in the AUC of the validation set. According to the results of this study, the random forest model may be slightly better than the stepwise regression model. But the nomogram based on the stepwise regression model was more convenient than the random forest model. This study recommends the nomogram for further external validating and updating.

### Implications

The predictive model presented here is mainly applicable to patients undergoing LIF who receive indwelling urinary catheters before surgery. It is not applicable to patients with cervical or thoracic degenerative disease, patients with nerve injury caused by fracture, or patients with cauda equina nerve injury before operation. It is also not applicable to patients with urological disease. For such patients, consultation with the urology department should be performed before extraction of the urinary catheter.

This model has high adaptability and low selectivity for the application environment. Data on the predictive variables are easily obtained without special environmental requirements. The most ideal application environment is a tertiary general hospital, and the technique of LIF is mature.

In the next external validation, it will be better to choose a comprehensive tertiary general hospital at which more than 300 LIF are performed in one year. In regard to model updating, it is better to include variables such as intraoperative anesthetic drugs, postoperative infusion volume, and 24-hour urine volume among the evaluated variables.

## Conclusion

In conclusion, we developed and internally validated a novel nomogram and a random forest model for predicting the risk of POUR after lumbar interbody fusion surgery. Both of the nomogram and the random forest model have high accuracy in this study. The nomogram need further external validation and update. Then the nomogram may help clinicians predict the probability of POUR for each patient individually so that targeted nursing and precautionary measures can then be taken to reduce the risk of POUR.

## Data Availability

The datasets generated and analysed during the current study are not publicly available due to ethical issue but are available from the corresponding author on reasonable request.

## List of Abbreviations

POUR: Postoperative urine retention
AUC: Area under the curve
ROC: Receiver operating characteristic
VAS: Visual analog scale
LIF: Lumbar interbody fusion
PLIF: Posterior lumbar interbody fusion
MIS: Minimally invasive surgery
MIS-TLIF: Minimally invasive surgery transforaminal lumbar interbody fusion
Endo-LIF: Endoscopic lumbar interbody fusion
TRIPOD: Transparent Reporting of a multivariable prediction model for Individual Prognosis Or Diagnosis
BMA: Bayesian model averaging
EPV: Events per variable
